# DEMENTIA CARE PARTNERS’ ANXIETY, DEPRESSION, AND BURDEN AT HOSPITAL DISCHARGE

**DOI:** 10.1093/geroni/igad104.0302

**Published:** 2023-12-21

**Authors:** Ashley Kuzmik

**Affiliations:** Pennsylvania State University, State College, Pennsylvania, United States

## Abstract

At the time of hospital discharge, family care partners are typically planning to deal with the increased care dependency and medically complex needs of the person living with dementia. Consequently, they experience compounded stress to their psychological wellbeing. This study examined patient and care partner factors associated with family care partners’ anxiety, depression, and burden when caring for patients with dementia at hospital discharge. The sample consisted of 434 patient and care partner dyads enrolled in the Family centered Function-focused Care (Fam-FFC) study. Multiple regression analyses were conducted to examine factors associated with care partners’ anxiety (Hospital Anxiety and Depression subscale anxiety; HADS-A), depression (Hospital Anxiety and Depression subscale depression; HADS-D), and burden (Short Form Zarit Burden Interview; ZBI-12). Care partner anxiety was associated with higher levels of patient behavioral and psychological symptoms of dementia (BPSD; β=.13; p = <.001). Care partner depression was associated with cohabitation (β=1.30; p = <.001), and patient factors including increased BPSD (β=.17; p = <.001) and lower function (β=-.03; p = <.001). Care partner burden was associated with care partner relationship with patient (adult child; β=-.27; p = .02) and higher patient BPSD (β=.67; p = <.001). Findings suggest the need to incorporate the functional and behavioral status and needs of the patient when preparing the care partner for discharge, which may be especially critical if the patient is discharged to the care partner’s residence.

